# Neurostimulation for Chronic Low Back Pain during Pregnancy: Implications for Child and Mother Safety

**DOI:** 10.3390/ijerph192315488

**Published:** 2022-11-22

**Authors:** Massimo Antonio Innamorato, Marco Cascella, Elena Giovanna Bignami, Paolo Perna, Emiliano Petrucci, Franco Marinangeli, Alessandro Vittori

**Affiliations:** 1 Department of Neuroscience, Pain Unit, Santa Maria delle Croci Hospital, AUSL Romagna, Viale Vincenzo Randi 5, 48121 Ravenna, Italy; 2 Department of Anesthesia and Critical Care, Istituto Nazionale Tumori—IRCCS, Fondazione Pascale, Via Mariano Semmola 53, 80131 Naples, Italy; 3 Anesthesiology, Critical Care and Pain Medicine Division, Department of Medicine and Surgery, University of Parma, 43121 Parma, Italy; 4 Department of Anesthesia and Intensive Care Unit, San Salvatore Academic Hospital of L’Aquila, Via Vetoio 48, 67100 L’Aquila, Italy; 5 Department of Anesthesiology, Intensive Care and Pain Treatment, University of L’Aquila, Piazzale Salvatore Tommasi 1, 67100 Coppito, Italy; 6 Department of Anesthesia and Critical Care, ARCO ROMA, Ospedale Pediatrico Bambino Gesù IRCCS, Piazza S. Onofrio 4, 00165 Rome, Italy

**Keywords:** pain, chronic pain, low back pain, child, mother, pregnancy, spinal cord stimulation, implantable pulse generator, pediatrics, neurostimulation

## Abstract

Pain therapy for low back pain in pregnancy is a very topical issue. In fact, it is necessary to balance the patient’s needs to control pain with the need to manage a pregnancy without negative effects on the fetus. We report a case of a 37-year-old woman with low back pain treated with neurostimulation before pregnancy. She described severe chronic low back pain unresponsive to pharmacologic treatments. We first implanted a subcutaneous stimulator into the patient, and then a definitive stimulator resulting in excellent pain control. The improvement in her quality of life allowed the woman to become pregnant. We decided to stop neurostimulation with the patient during pregnancy. The patient completed her pregnancy without complications and the baby was born healthy. During the pregnancy, the woman took only paracetamol when needed. However, this painful symptomatology, completely anecdotal, is not attributable solely to the previous spine problem but probably also to the changes occurring during pregnancy. At the end of pregnancy, the neurostimulator was reactivated without any discomfort for the patient, who is now pain free. This case report provides a first line of evidence of a possible treatment of low back pain in women intending to become pregnant, with risk-free management for both the patient and the child.

## 1. Introduction

Chronic low back pain represents a health, social and economic problem [1]. In fact, it is increasingly evident that this pathology, often without a clear and defined etiology, is a condition that has very high costs in terms of healthcare, lost working days and a reduction in the ability of individuals to function [2,3,4]. The new vision of chronic pain as a biopsychosocial phenomenon finds a paradigm in low back pain [5]. Despite being such a widespread condition not only in numerical terms but also by geographical spread, there is no univocity of treatment, often with guidelines that are not always in agreement [6]. However, there is consensus in recognizing chronic pain as a pathology in its own right, which has lost its protective significance (as occurs in acute pain), with relevant neurological modifications [7,8,9]. Several studies, mainly carried out in vitro and concerning possible treatments for degenerative diseases of the nervous system or cell damage, have shown how nerve cells are sensitive to electromagnetic fields and direct their growth along the lines of the field itself. Regarding the influence of electromagnetic fields in vivo on pregnancy, there is no univocal opinion [10,11]. It is reported that exposure to an electromagnetic field greater than 2 mG (0.2 microT) and using an electric thermal blanket during pregnancy is not related to a greater risk of underweight newborns or newborns with intrauterine growth retardation [12]. Mahran et al. conducted a study on the exposure of pregnant women to different electromagnetic fields produced by common household appliances and did not observe any type of effect on pregnancy or the fetus, but nevertheless recommended that women limit such exposure during this period as a precautionary measure [13]. Studies on the effects of exposure to electromagnetic fields of women in the first trimester of pregnancy have found that exposures of >16 mG increase the risk of spontaneous abortion. This type of relationship is stronger for early miscarriages (before 10 weeks) [14,15,16]. Comparing several studies in which pregnant women were subjected to electrical cardioversions or suffered accidental electric shocks, it was found that these events did not lead to problems with the pregnancy or the fetus [17,18,19]. Based on this data, and considering the much smaller amount of electrical and electromagnetic forces generated by spinal cord stimulation (SCS), it can be assumed that low voltages are reasonably safe. Furthermore, recharging the device produces electromotive forces that are much stronger than the switched system itself, but despite this, the vertebrae, pelvis, and tissues produce an insulating effect, especially when the implantable pulse generator (IPG) is implanted in the gluteal region. There is no scientific evidence to support the hypothesis that neurostimulation can stimulate uterine contractions [20].

Possible effects of neurostimulation during pregnancy are:

- On the fetus: teratogenicity of fetal malformations.
- On women and pregnancy: abortion, premature birth, irritation and ulceration of the skin stretched on the battery, obstetric or anesthetic difficulties or complications, pain at the electrode or implant site.
- On the device: migration of the electrode, depletion of the battery, stretching of the extension following expansion of the abdomen [13,21].

The first trimester is the most critical period in which it is recommended to avoid electrical stimulation [13]. The electromotive forces produced by neurostimulation are unlikely to reach the developing fetus.

Chronic pain is a difficult field in terms of both research and patient care [22]. In fact, treatments integrating invasive methods and advanced pharmacological therapies are often required to obtain not only adequate pain control but also functional recovery [23]. The most important goal of chronic pain management is not pain control alone, but full functional recovery and improved quality of life. All this is even further complicated when it is associated with pregnancy which makes therapeutic choices very limited, even for medico-legal reasons [24]. In addition, low back pain is a fairly common condition during pregnancy (with a prevalence ranging from 24 to 90%) [25,26]. Low back pain in pregnant women can have important sequelae in her personal life and quality of life, for example, by altering the quality and duration of sleep [27,28,29]. It is a research field that still has some unclear sides, especially regarding etiology and treatment. However, among the established risk factors, a history of low back pain prior to pregnancy appears to be one of the most important [26,30]. Despite such a significant prevalence of the repercussions impacting the quality of life, less than 50% of pregnant women with low back pain receive adequate (pharmacological and/or invasive) therapies [29,31]. Neurostimulation can be an important alternative to drug treatment in a failed back surgery syndrome (FBSS) [32,33,34]. In fact, neurostimulation involves the insertion of electrical devices capable of blocking the painful signal or modulating it [35]. Neurostimulation plays an extremely important role when drug therapy fails to control pain [35]. In fact, the FBSS is a difficult-to-treat condition in which drug treatment alone may not be effective [36].

This case report shows how through adequate pain control it is possible to obtain a complete functional recovery.

## 2. First Clinical Evaluation and Background

The clinical case presented below concerns a female patient aged 35 at the time of delivery. The medical history of the patient reports arthrodesis for thoracolumbar scoliosis (stabilization with Harrington in 1997), with removal of the broken bar and permanent Luque threads (removal of the means of fixation in 2009). In 2015, she underwent L5 and S1 laminectomy surgery with prosthetic replacement of the L5-S1 interbody disc and posteriorly instrumented arthrodesis.

After surgery, persistence of lumbar pain radiating to the lower limbs, especially in the L5-S1 area on the left, with a numerating rating scale (NRS) score 6 was observed. The pain of medium intensity was constant with peaks occurring throughout the day. About a year after the neurosurgery, she received a course of 10 paravertebral corticosteroid infiltrations from which she gained temporary benefit. Unfortunately, we have no clinical reports of this treatment because it was performed at another center and the patient did not provide us with any documentation. On 4 October 2016, the patient was seen at the Pain Unit of Ravenna. The patient’s therapy consisted of the following: duloxetine 60 mg/day, palmitoylethanolamide (PEA) 600 mg/day, association of oxycodone + naloxone 5 mg + 2.5 mg three times a day, ibuprofen 400 mg as needed, with a multimodal approach [23,37,38]. However, this pharmacological treatment did not provide adequate pain control and decreased the patient’s quality of life. In fact, the patient presented with a 10 m claudication and had to alternate between crutches and wheelchair to be able to move around. This functional limitation greatly affected her social life, effectively preventing the possibility of becoming pregnant. In fact, chronic low back pain is a biopsychosocial disease [5,39].

The patient certainly had a desire to become pregnant, but it was impossible not only to start a pregnancy, but also to suspend the very demanding pharmacological therapy.

## 3. Case Presentation

**Figure 1:**
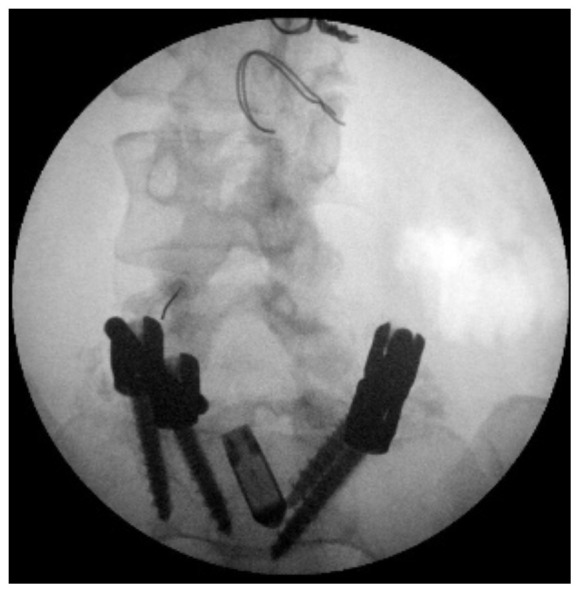
X-ray image of the L4-L5 facet joints before implantation.

In the first instance, she underwent an infiltrative cycle of the apophyseal facets of the left L3-L4 and L4-L5 on 17 and 24–31 October 2016 (on each occasion the bilateral L4-L5 lumbar joint facets were infiltrated with dexamethasone 16 mg, ropivacaine 5 mg 0.2%, triamcinolone 40 mg, which produced little benefit, numerating score scale score (NRS) 7 (Figure 1)). The patient’s condition was so poor that it would have been impossible to plan an effective physiotherapy rehabilitation intervention as the patient experienced pain too intense to do so. Indication for spinal cord stimulation (SCS) was then given [40,41].

**Figure 2:**
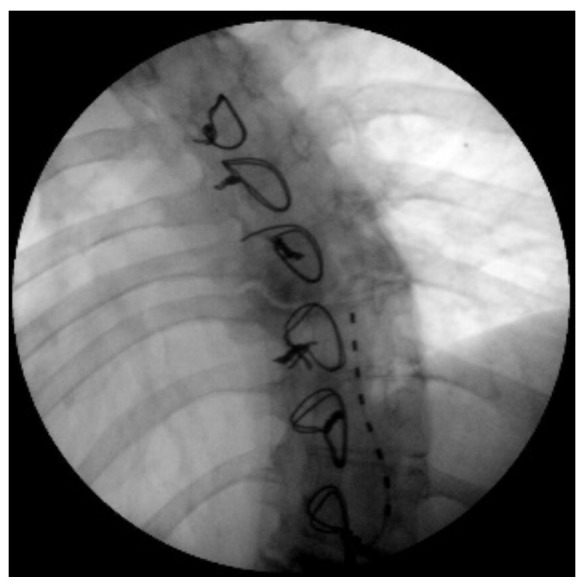
X-ray image of the subcutaneous catheter positioned between D8 and D10.

**Figure 3:**
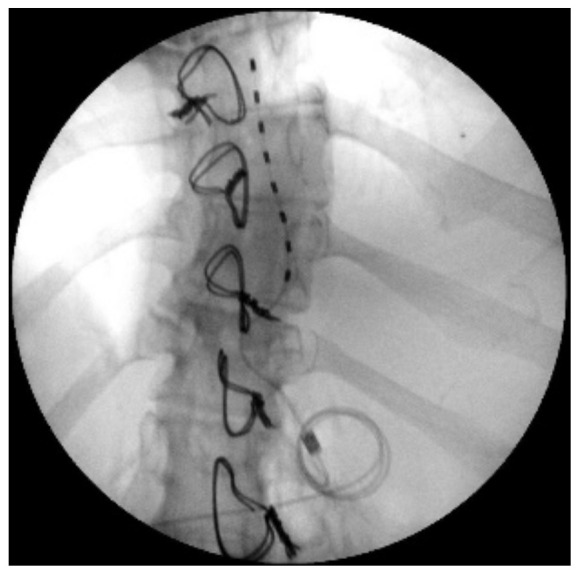
X-ray image of loop for subcutaneous fixation.

**Figure 4:**
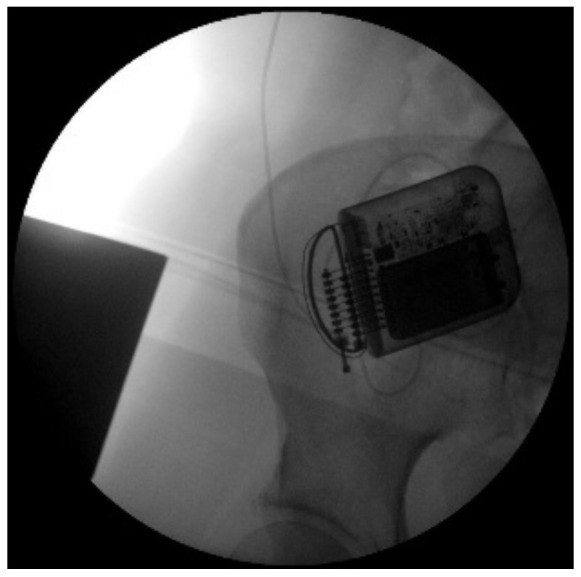
X-ray image of the neurostimulator in right iliac fossa.

The indication was then given for the placement of a peridural medullary lead for high frequency spinal cord stimulation (10,000 Hz) Nevro Senza^TM^ (Italy) on 24 January 2017 as the first interventional step (pulse width 30 µs; current delivered 3 mA; charging time of 40 min every 24 h). However, the intervention was technically impractical due to the anatomical conformation of the column, the presence of means of synthesis and the results of previous interventions not permitting epidural access. Therefore, during the operation, it was decided to position the lead in the paravertebral subcutaneous space. This choice proved to be feasible and the electrode was implanted (Figure 2). During the three-month follow-up period (algological visit every two weeks), the patient reported good control of the symptoms (with resumption of normal sexual life) due to which it was decided to proceed to the second interventional step and the subcutaneous positioning of the definitive implant [42]. In the second procedure, the generator was implanted in a subcutaneous pocket packed in the abdomen (right side) on 21 February 2017 (Figure 3 and Figure 4). Neurostimulation reduced pain, allowing a progressive reduction of drug therapy until its total suspension two months after the implant. About three months after the implant, the patient informed the pain therapy service that she was pregnant, raising questions about the possible effects of neurostimulation on the fetus. After extensive literature searches and discussions within our team, as well as consultation with experts, we explained to the patient that the findings in the literature were often contradictory and thus did not provide any clear conclusions [43,44,45,46,47]. In fact, there are no absolute certainties dispelling any possible doubt about the health of the fetus [47].

Prudently, the patient chose to suspend the stimulation. The timing of the suspension of the stimulation was at the end of the third month of gestation. This timing is due to the time needed to diagnose pregnancy, the time needed to develop a therapeutic strategy, and finally, but much more importantly, the time needed by the patient to make a decision based on the information received from the physicians. However, the pregnancy proceeded normally, with no obstetric problems. Pain control was optimal as the patient took paracetamol 1000 mg orally when needed. It should be emphasized that the painful episodes that required paracetamol intake cannot be attributed solely to the underlying pathology, but could also be a consequence of the change in load on the spine due to pregnancy. A caesarean section was performed at the 37th week of gestation in January 2018 and the baby was born healthy with Apgar score of 9/10 at birth, 10/10 at 1 min; weight 3300 g and 50 cm. From March 2018 to today, the patient maintains excellent pain control with no problems in the operation of the implant.

## 4. Discussion

The orientation that several authors agree on is to use an upper lumbar or thoracic medullary access for the positioning of the lead for women of childbearing age and, if possible, to place the implantable pulse generator (IPG) in the gluteal region rather than the abdominal one. The aim of these two suggestions is to not compromise any anesthetic maneuvers such as the placement of an epidural catheter for analgesic delivery or the choice of performing a subarachnoid locoregional anesthesia for a caesarean section, reducing the risk of stretching the extension (with growth abdominal diameter) and not creating any surgical difficulties.

It has been highlighted by several authors that subarachnoid anesthesia does not involve any additional risk for patients with a neurostimulation implant, provided that the puncture is situated in a region below the device. Even epidural anesthesia and catheter placement were not related to migration problems of the already implanted lead, with the usual foresight of keeping lower than the device when positioning the epidural catheter. However, maximum care must be taken during the administration of anesthetic boluses to ensure absolute sterility, as the risk of implant infections, albeit rare, is present. In pregnant women with an implant, the choice of elective caesarean section is often considered as the only option. Some authors justify this choice by explaining that, especially in regard to the sacral stimulators, they could be damaged by the thrusts of the woman in the gynecological position who rests all her weight and thrusts on the buttocks. However, this is not supported by the evidence. Studies on malfunctioning report a higher frequency in cases of caesarean section (38%) than in vaginal deliveries (25%) [48]. Therefore, the indication for a caesarean section should only concern obstetric problems and not just the fact of involving a neurostimulator. In our case, the caesarean section was indicated not due to the presence of the electro stimulator, but on the basis of two other reasons. The first, of an obstetric nature, provided for the impossibility of risk-free labor given the particular situation of the patient’s spine. It should be mentioned that obstetric surgery is one of the fields subject to the greatest number of legal medical complaints in Italy [24]. The second reason was the choice of the patient. In fact, in Italy, one of the indications for caesarean section is the so-called “self-determination”, that is, the choice of the pregnant woman who prefers to undergo a caesarean section rather than face a difficult and dangerous labor. The implantation of the stimulator created a means of avoiding potentially teratogenic drugs, allowing the onset of pregnancy for two reasons. The first undoubtedly concerns the improvement in the quality of life, with the resumption of normal personal life and a normal gait. The second reason was that of the suspension of risky drugs, necessary before implantation for pain control, albeit inadequate. It should be emphasized that the patient, after the implantation of the stimulator, no longer used drugs, except for paracetamol as needed, preserving the health of the fetus and giving birth to a healthy child.

A special mention should be given to the rehabilitation option which is also important in chronic low back pain treatment [49]. We offered the patient physiotherapy and rehabilitation treatments which were also indicated [50]. However, the patient’s denial of such treatments was due to her very poor quality of life which prevented her from undertaking any physical activity. Therefore, although aware of the extreme importance of a multidisciplinary treatment, we opted for an attack therapy that could quickly restore an acceptable quality of life. In addition, the evidence of rehabilitation is there, but it is not of high quality, and we were not allowed to waste any more time on a patient who needed immediate improvement [51].

## 5. Conclusions

An important aspect highlighted by the clinical case described is the issue of using drugs with a possible teratogenic effect. Many drugs commonly used for pain control, such as anticonvulsants, antidepressants, opioids and NMDA antagonists, result in diverse side effects on the fetus and fall into category C of Food and Drug Administration classification (FDA), i.e., “studies carried out on animals have shown adverse effects on the fetus, but there are no adequate control studies in humans and the potential benefits guarantee the use of the drug in pregnant women despite a potential risk“. Patients who have to undergo treatments with these drugs usually choose to use contraceptives so as to avoid pregnancy and risks to the fetus. The patient described took advantage of a treatment-free window to successfully attempt conception. This case report presents a first line of evidence of the possibility to treat low back pain in women intending to become pregnant, with risk-free management for the patient and the child.

## Data Availability

The data can be requested from the corresponding author for a reasonable purpose.
